# Mobile phone addiction is associated with impaired cognitive reappraisal and expressive suppression of negative emotion

**DOI:** 10.3389/fpsyt.2022.988314

**Published:** 2022-09-20

**Authors:** Jin Liu, Zhaojun Xu, Lili Zhu, Renliying Xu, Zhaocai Jiang

**Affiliations:** ^1^Department of Psychology, School of Educational Science, Ludong University, Yantai, China; ^2^Institute for Education and Treatment of Problematic Youth, Ludong University, Yantai, China; ^3^Department of Moral Education, Yantai No. 3 Middle School, Yantai, China

**Keywords:** mobile phone addiction, emotion regulation, cognitive reappraisal, expressive suppression, addiction

## Abstract

Previous studies have demonstrated people characterized by mobile phone addiction (MPA) are more prone to emotion regulation difficulties. However, no study has tested the effectiveness of their emotion regulation strategies in experimental conditions. In the present study, by instructing the MPA and control groups to regulate negative emotion through cognitive reappraisal (CR) or expressive suppression (ES), we compared their emotional states in the emotional visual search task after watching a negative emotion evoked video. A multi-factor mixed design of 2(group: MPA/control)×2(emotion regulation strategy: CR/ES)×3(image type: positive expression/negative expression/neutral expression) was conducted. We found the MPA group recognized the negative expression faster than control group after both emotion regulation strategies, indicating ES and CR were both impaired for MPA. The implications of these results were further discussed.

## Introduction

With the rapid development of information technology, mobile phone has become one of the most influential technologies in people's life in China. Mobile phone addiction (MPA) is characterized by one's inability to control their mobile phone use and thus leads to physiological and psychological discomforts, mood changes as well as negative life consequences (1, 2). According to the affective processing model of negative reinforcement, an important reason for the maintenance of addiction is that addicts can't deal with the negative emotions accompanied with addiction (3). Similarly, some researchers have proposed that it is precisely because of individuals' deficiency in regulating negative emotions that MPA continues to maintain (4). Moreover, recent empirical studies demonstrated people characterized by MPA were more prone to emotion regulation difficulties (5, 6). However, to date, no study has explored the effectiveness of their emotion regulation strategies in experimental conditions. Thus, the current study aims to test whether mobile phone addicts present impairments in specific emotion regulation strategies when they are instructed to regulate emotions during watching a negative emotion evoked video.

There are different emotion regulation strategies which people use to decrease/increase emotional response tendencies (7). Among them, cognitive reappraisal (CR) and expressive suppression (ES) are two most studied ones (8–10). CR occurs early in the emotion-generating process and refers to having benign or positive interpretations in stressful situations to reduce negative emotions (10, 11). ES involves inhibiting the outward expression of emotion (e. g., facial expression and gesture) after it has already been generated (7, 11). Although both strategies are effective, there seemed to be difference for people addicted to different substances. For example, opioid addicts tend to prefer ES, while methamphetamine addicts usually use CR (12).

In fact, several studies have explored the effectiveness of CR in addicts but the results seemed to be inconsistent. Baicy found compared with healthy controls, methamphetamine dependents demonstrated a deficit in the left inferior frontal cortex during emotion regulation with CR (13). Albein-Urios et al. found cocaine dependents showed decreased posterior cingulate cortex, insula and fusiform gyrus activation during CR (14). However, another study found there was no significant difference in emotional response to pictorial scenarios between heavy smokers and non-smokers after reappraising, indicating nicotine addicts do not have impairments in deliberate CR (15). Moreover, until now, no study has explored the effectiveness of ES in addicts. Therefore, in the present study, by instructing the MPA and control groups to regulate emotions when watching a negative emotion evoked video, we aimed to test whether mobile phone addicts presented impairments in CR, ES, or both. We hypothesized:

**H1**: Mobile phone addicts may present impairments in CR. That is, compared with healthy controls, mobile phone addicts may experience more negative emotions after emotion regulation with CR.

**H2**: Mobile phone addicts may present impairments in ES. That is, compared with healthy controls, mobile phone addicts may experience more negative emotions after emotion regulation with ES.

## Materials and methods

### Participants

A total of 489 participants (aging 16–21, age M ± SD = 19.21 ± 0.72) were randomly invited through campus advertisement in Ludong University and completed Mobile Phone Addiction Index (MPAI). The MPAI is a 17-item 5 point Likert scale developed based on Young's Internet addiction scale and has good reliability and construct validity in Chinese adolescents (16, 17). In the current sample, the Cronbach's α was 0.89 for the MPAI. According to previous study (17), 70 of the participants (including 34 females and 36 males, age M ± SD = 18.93 ± 0.86) scoring no < 40 in MPAI were classified as the MPA group. The control group, including 70 participants, was selected from the remaining scoring < 40 with matched age and sex (age M ± SD = 19.30 ± 1.36). All subjects completed SCL-90 and none of them had mental illness or negative emotion distress. Then, each group was randomly divided into 2 subgroups according to the guidance of emotion regulation strategy: CR subgroup and ES subgroup. The study was approved and supervised by the Institutional Review Board, sponsored by the China Association for Science and Technology (CAST) and the Ministry of Health of the People's Republic of China. All subjects gave their informed consent for inclusion before they participated in the study. Due to the sensitive nature of the questions we asked, all subjects were assured no personal information would be disclosed during or after the study.

### Experimental design and procedure

**Figure 1:**
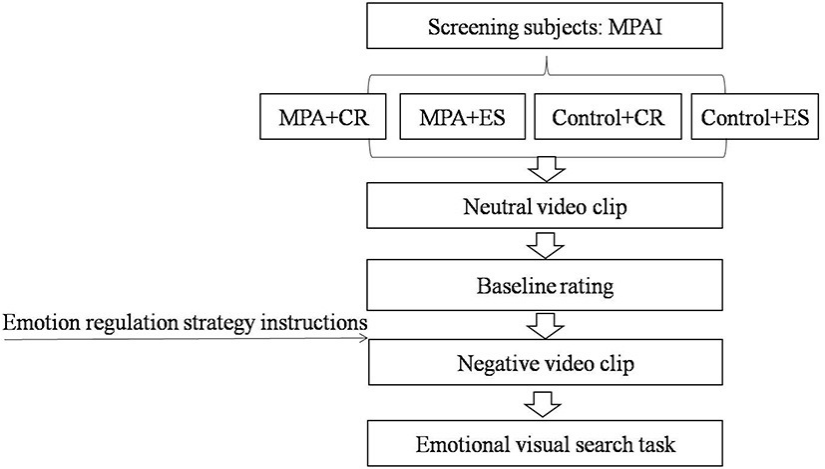
Experiment procedure. MPAI, Mobile Phone Addiction Index; MPA, Mobile phone addiction; CR, Cognitive reappraisal; ES, Expressive suppression.

A multi-factor mixed design of 2(group: MPA/control)×2(emotion regulation strategy: CR/ES)×3(image type: positive expression/negative expression/neutral expression) was adopted. Group and emotion regulation strategy were between-subject variables, while the image type was the within-subject variable. Experimental procedure was shown in Figure 1. To avoid the interference of baseline emotion states, we firstly asked all participants to watch a 4-min neutral video clip and rated their baseline emotion states. Then, participants were given instructions of different emotion regulation strategies according to their subgroups as previous study demonstrated (8). For the CR subgroup, the instruction was as follows: Next, you will see a video clip. The video has an artificially exaggerated effect and does not equate to reality. During watching, when you feel emotions, you should try to keep an objective attitude toward video as much as possible. For the ES subgroup, the instruction was as follows: Next, you will see a video clip. During watching, when you feel emotions, you should not show it. Try to cover up your feelings and do not let others see it. Then, the negative emotion video material was presented. After that, all participants completed the emotional visual search task.

### Emotion evoked video materials

We adopted video clips of “Tangshan earthquake” and “March of the Penguins” as the negative and neutral emotion evoked video materials respectively. Both video clips lasted for 4 min. To test their effectiveness in the current study, we recruited another 50 students (age M ± SD = 18.74 ± 0.88) randomly to assess the valence of emotions evoked by these two video clips from 1 to 10. 1 = very positive, 5 = neutral, 10 = very negative. Results indicated video clips of “Tangshan earthquake” and “March of the Penguins” could effectively induce negative and neutral emotions respectively (for “Tangshan earthquake,” M ± SD = 7.68±1.00; for “March of the Penguins,” M ± SD = 4.68±1.83; *t* = 10.45, *p* < 0.001, *d* = 1.45).

### Emotional visual search task

**Figure 2:**
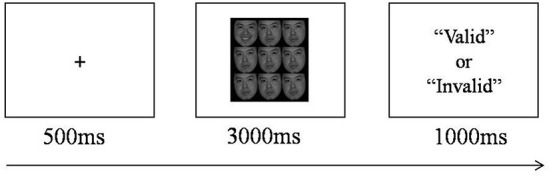
The timelines of the emotional visual search task (single trial).

A total of 6 facial expression pictures (including happy, angry and neutral pictures of a male and a female) were selected from the Chinese Facial Affective Picture System (18). They were made into a matrix with nine face images in format 3 × 3. Each matrix has a width of 9.98 cm and a height of 10.69 cm, with pixel size 283 × 303 and resolution 72. During the emotional visual search task, two types of face matrix (with or without target stimulus) were presented. In the matrix without target stimulus, all 9 pictures were neutral facial expression. While the matrix with target stimulus consisted of 8 neutral facial pictures and a target stimulus (i e., happy or angry expression) randomly presented at any position of the matrix. A total of 72 trials were included. Thirty six trials were presented without target stimulus and the other 36 trials with target stimulus (half happy and half angry). The task was conducted in E-prime 3.0 (Figure 2). A “+” fixation was firstly presented on the screen for 500 ms. Then, the face expression matrix was presented for 3000 ms. Subjects were required to judge whether the expression pictures in the matrix were exactly the same within 3000 ms. If they were exactly the same, press the “J” key. If not, press the “F” key. After that, there would be a feedback (i e., “valid” or “invalid”) for 1000 ms. If the participant's react was within 3000 ms, the feedback would be “valid.” If the participant did not press any key within 3000 ms, the feedback would be “invalid.”

### Data analysis

The SPSS 21.0 was used for data analysis. One-way ANOVA was run to test group difference in baseline emotion states. The average response times (RTs) in the emotional visual search task were calculated with all unresponsive trials deleted. Shapiro-Wilk Test was firstly used to test the normality of average RTs. To determine the difference in average RTs, a repeated measurement ANOVA of 2(group: MPA/control)×2(emotion regulation strategy: CR/ES)×3(image type: positive expression/negative expression/neutral expression) was conducted with group and emotion regulation strategy as the between-subject factors and image type as the within-subject factor. Bonferroni posttest was employed for *post hoc* test.

## Results

**Figure 3:**
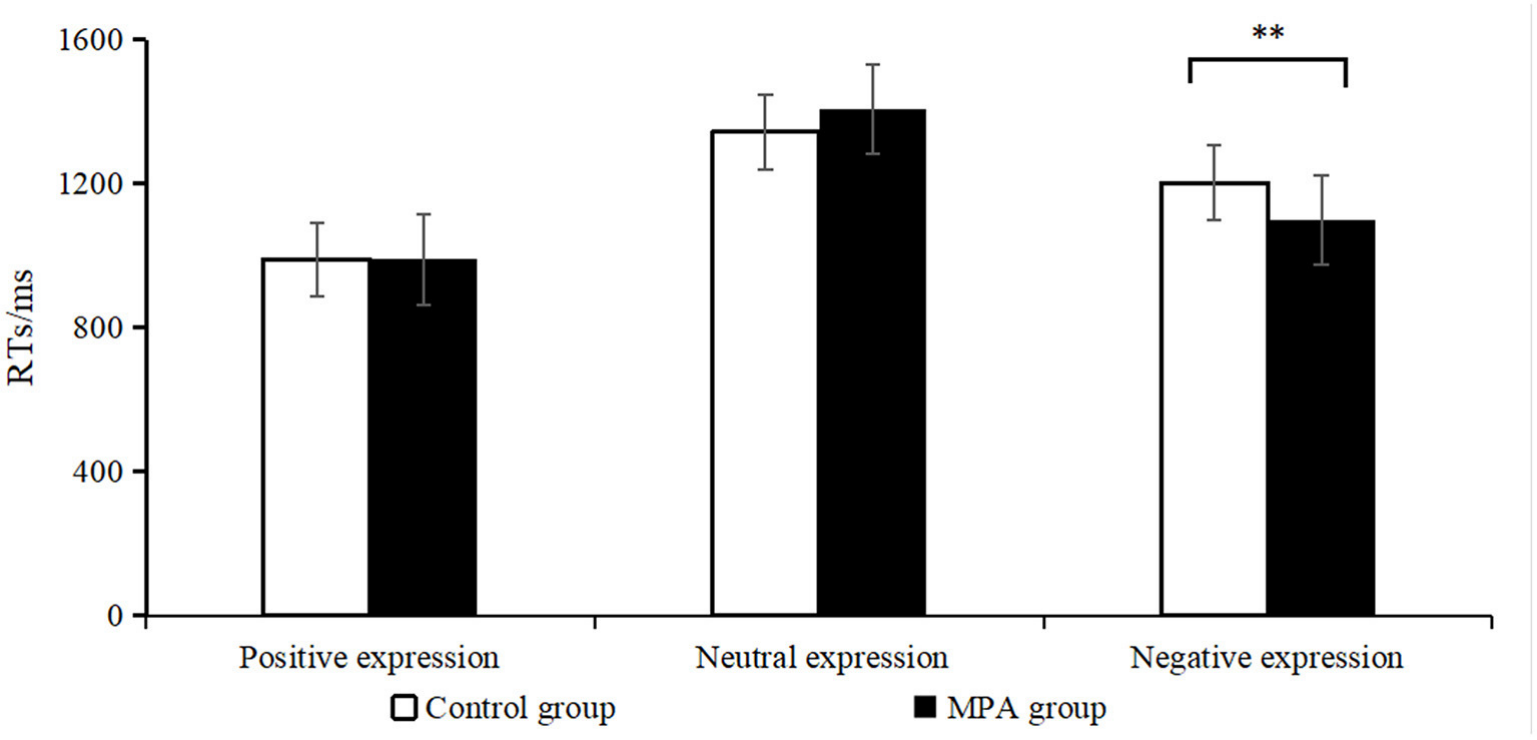
Comparison of average RTs in the emotional visual search task. MPA, mobile phone addiction. The error bar in this figure means standard error (SE). **p < 0.01.

To avoid the interference of baseline emotion states, we firstly asked all participants to watch a 4-min neutral video clip and rated their baseline emotion states. There was no significant difference in their baseline emotional states after watching neutral video clip, *F*_(3)_ = 1.05, *p* = 0.37, η^2^ = 0.02 (see Supplementary Table 1). As for the emotional visual search task, Shapiro-Wilk Test showed the data of average RTs was normally distributed (*p* > 0.05 for all subgroups). Table 1 presented the average RTs for different groups and the repeated measurement ANOVA was shown in Supplementary Table 2. The main effect of group was not significant, *F*_(1)_ = 0.26, *p* = 0.61, η^2^ = 0.002. However, the main effect of emotion regulation strategy was significant, *F*_(1)_ = 4.58, *p* = 0.03, η^2^ = 0.03. The average RTs for CR was faster than that of ES (1142.02 ± 19.45 vs. 1200.91 ± 19.45). Additionally, the main effect of image type was significant, *F*_(2)_ = 231.15, *p* < 0.001, η^2^ = 0.63. The average RTs for positive expression was faster than that of negative expression, which was faster than that of neutral expression (989.30 ± 13.88 vs. 1150.34 ± 18.40 vs. 1374.77 ± 19.34). More importantly, we found the interaction between image type and group was significant, *F*_(2)_ = 10.80, *p* < 0.001, η^2^ = 0.07. A simple effect test was further conducted (see Figure 3). For the neutral or positive expression, there was no significant difference between the two groups. However, for the negative expression, the MPA group responded significantly faster than the control group (1098.6 ± 26.02 vs. 1202.08 ± 26.02, *t* = 2.81, *p* = 0.006, *d* = 0.48, Bonferroni corrected).

**Table 1:** The average RTs for different groups.

**Image type**	**Control + CR**	**Control + ES**	**MPA + CR**	**MPA + ES**
Positive expression	942.77 ± 190.46	1036.51 ± 210.73	967.49 ± 105.25	1010.41 ± 110.96
Neutral expression	1320.10 ± 257.70	1367.06 ± 275.70	1344.38 ± 188.93	1467.56 ± 159.52
Negative expression	1190.74 ± 248.91	1213.42 ± 253.07	1086.65 ± 170.00	1110.53 ± 192.11

Control + CR, control group with cognitive reappraisal; Control + ES, control group with expressive suppression; MPA + CR, mobile phone addiction group with cognitive reappraisal; MPA + ES, mobile phone addiction group with expressive suppression.

## Discussion

In this experiment, we found the main effect of emotional regulation strategy was significant and the average RTs of CR was faster than ES. Yet, the effect size was 0.03 and relatively small. This finding was consistent with previous studies demonstrating that ES was associated with reduced judgment speed (7) and reasoning ability (19). This could be explained by the fact that ES, which is used after emotion has already been generated, triggers stronger physiological arousal and requires continuous cognitive efforts to inhibit emotion (7–11). On the other hand, some researchers also claimed that slower reaction speed does not necessarily represent poor emotion regulation (7, 9, 19). Furthermore, our study only included RT as the dependent variable, without additional relevant index (such as the accuracy). Combined all these, although the main effect of emotional regulation strategy was significant in our study, we don't want to claim that our results definitely support the strategy of CR is more effective than ES. Additionally, we demonstrated the main effect of image type and the effect size was large. The average RTs for positive expression was faster than that of negative expression, which was faster than that of neutral expression. The visual expression search task required subjects to search for the positive/negative expression in the neutral background as soon as possible. Our findings is consistent with previous studies demonstrating people have attention bias to positive information (20).

More importantly, we found significant interaction effect between image type and group, with a medium effect size. Mobile phone addicts recognized the negative expression faster than controls after emotion regulation strategies. According to the mood-congruent effect (21), positive expression would be detected faster for individuals in the positive emotion state and negative expression would be detected faster for individuals in the negative emotion state as well. Thus, the accelerated detection of negative expression for mobile phone addicts indicated their negative emotions were stronger than healthy controls after emotion regulation strategies. Considering there was no significant difference in their baseline emotional states, the most likely explanation for the result may be that ES and CR in MPA were both impaired. For ES, to our knowledge, this is the first study showing impaired ES in addicts. As for CR, this is in accord with previous studies showing impaired CR in cocaine (14) and methamphetamine dependents (13). However, our result is inconsistent with Wu et al. (15) demonstrating heavy smokers performed comparably to non-smokers in regulating emotional response *via* CR. One possible reason may be that in that study, there was a reappraisal practice to ensure nicotine addicts completely master the deliberate reappraisal strategy before the experiment (15). Thus, whether CR of mobile phone addicts can be comparable to healthy controls after a reappraisal training needs to be reexamined in future research.

Mobile phone addicts are characterized by impulsivity (1) and lack of inhibition control (22). On the other hand, inhibition control deficiency is associated with emotion regulation difficulties in children (23), adolescents (24) and adults (25). The development of emotion regulation ability depends on the maturity of inhibition control (23). Considering MPA and emotional regulation difficulties share common inhibition control deficiency, therefore, we believe the impaired emotion regulation in MPA might be attributed in part to inhibition control deficiency. In addition, emotion regulation, especially CR, depends on fine cognitive ability. People need more cognitive resources to reinterpret events or overturn the original interpretation when CR is adopted (26). However, MPA was associated with cognitive impairments, such as reduced available cognitive capacity (27), worse cognitive control and deteriorating cognitive flexibility (28). Combined with all these studies, we speculate that the impaired emotion regulation might be attributed to inhibition control deficiency or the cognitive impairments in mobile phone addicts, which needs yet to be proved by following studies.

To sum up, in the present study, we found mobile phone addicts detected the negative expression faster than healthy controls after ES and CR, indicating emotion regulation strategies were both impaired. Some limitations of this study should be noted. Firstly, during the emotional visual search task in this study, we only recorded RT as the dependent variable. In fact, our results would be more convincing if additional relevant index (such as the accuracy) could be integrated together. Therefore, we recommend the accuracy should be taken into full account for future studies adopting this task. Secondly, the cross-sectional design could not confirm causal relationships. Actually, causal pathways from MPA to impaired emotion regulation and from impaired emotion regulation to MPA are both theoretically plausible (5). Thus, future longitudinal studies are needed to identify their causal relationships. Additionally, our study only provided behavioral level of evidence. Whether impaired emotion regulation in MPA is accompanied with abnormal structural or functional changes in prefrontal cortex, anterior cingulate cortex or other brain regions is worth studying in future studies.

## Data availability statement

The raw data supporting the conclusions of this article will be made available by the authors, without undue reservation.

## Ethics statement

The studies involving human participants were reviewed and approved by the Ludong University Institutional Review Board. Written informed consent to participate in this study was provided by the participants' legal guardian/next of kin.

## Author contributions

JL collected the data and wrote the initial draft of the manuscript. ZX contributed to writing some elements of the manuscript. LZ and RX performed the statistical analyses. ZJ contributed to conceptualization and writing—review and editing. All authors contributed to the theorizing and conceptualization of the study, revisions of the manuscript, and read and approved the final manuscript.

## Funding

This work was supported by the Science and Technology Support Plan for Youth Innovation of Universities in Shandong Province (Grant number 2021RW002).

## Conflict of interest

The authors declare that the research was conducted in the absence of any commercial or financial relationships that could be construed as a potential conflict of interest.

## Publisher's note

All claims expressed in this article are solely those of the authors and do not necessarily represent those of their affiliated organizations, or those of the publisher, the editors and the reviewers. Any product that may be evaluated in this article, or claim that may be made by its manufacturer, is not guaranteed or endorsed by the publisher.
