# Supplementary material for: Mobile phone addiction is associated with impaired cognitive reappraisal and expressive suppression of negative emotion
Source: Front Psychiatry. 2022 Sep 20;13:988314. doi: 10.3389/fpsyt.2022.988314 (PMC9531033; doi:10.3389/fpsyt.2022.988314)
Supplement: Supplementary file 1 [file Data_Sheet_1.DOCX]

| Table 1. Baseline emotion states of the 4 groups | | | | | | | |
| --- | --- | --- | --- | --- | --- | --- | --- |
|  | Control+CR | Control+ES | MPA+CR | MPA+ES | *F* | *p* | *η^2^* |
| March of the Penguins | 5.94±1.64 | 6.14±1.75 | 5.51±1.56 | 5.66±1.55 | 1.05 | 0.37 | 0.02 |

Note: Control+CR = control group with cognitive reappraisal; Control+ES = control group with expressive suppression; MPA+CR = mobile phone addiction group with cognitive reappraisal; MPA+ES = mobile phone addiction group with expressive suppression.

|  | | | | | |  |
| --- | --- | --- | --- | --- | --- | --- |
| Table 2. The repeated measurement ANOVA of average RTs | | | | | |  |
|  |  | *df* | *F* | *p* | *η^2^* | |
| Within |  |  |  |  |  | |
| subjects |  |  |  |  |  | |
|  | Image type | 2 | 231.15 | < 0.001 | 0.63 | |
|  | Image type×Group | 2 | 10.8 | < 0.001 | 0.07 | |
|  | Image type×Emotion regulation strategy | 2 | 1.58 | 0.21 | 0.01 | |
|  | Image type×Emotion regulation strategy×Group | 2 | 1.57 | 0.21 | 0.01 | |
|  |  |  |  |  |  | |
|  | Error | 272 |  |  |  | |
| Between subjects |  |  |  |  |  | |
|  | Group | 1 | 0.26 | 0.61 | 0.002 | |
|  | Emotion regulation strategy | 1 | 4.58 | 0.03 | 0.03 | |
|  | Group×Emotion regulation strategy | 1 | 0.03 | 0.87 | 0.0001 | |
|  | Error | 136 |  |  |  | |
